# In silico screening of some compounds derived from the desert medicinal plant *Rhazya stricta* for the potential treatment of COVID-19

**DOI:** 10.1038/s41598-022-15288-2

**Published:** 2022-07-01

**Authors:** Nabih A. Baeshen, Abdulaziz O. Albeshri, Naseebh N. Baeshen, Roba Attar, Alaa Karkashan, Basma Abbas, Thamer A. Bouback, Abdullah A. Aljaddawi, Mohammed Y. Refai, Hayam S. Abdelkader, Abdullah Al Tamim, Abdullah Alowaifeer, Firoz Ahmed, Mohammed N. Baeshen

**Affiliations:** 1 Department of Biological Sciences, Faculty of Science, King Abdulaziz University, Jeddah, Saudi Arabia; 2 Department of Biology, College of Sciences and Arts, University of Jeddah, Khulais Campus, Jeddah, Saudi Arabia; 3 Department of Biology, College of Science, University of Jeddah, Jeddah, Saudi Arabia; 4 Department of Biochemistry, College of Science, University of Jeddah, Jeddah, Saudi Arabia; 5 Reference Laboratory for Food Chemistry, Saudi Food & Drug Authority (SFDA), Riyadh, Saudi Arabia

**Keywords:** Health care, Medical research, Biochemistry, Diseases, Infectious diseases, Viral infection, Microbiology, Pathogens, Virology, Drug discovery, Drug screening, Target identification, Target validation

## Abstract

The latest coronavirus pandemic (SARS-CoV-2) poses an exceptional threat to human health and society worldwide. The coronavirus (SARS-CoV-2) spike (S) protein, which is required for viral–host cell penetration, might be considered a promising and suitable target for treatment. In this study, we utilized the nonalkaloid fraction of the medicinal plant *Rhazya stricta* to computationally investigate its antiviral activity against SARS-CoV-2. Molecular docking and molecular dynamics simulations were the main tools used to examine the binding interactions of the compounds isolated by HPLC analysis. Ceftazidime was utilized as a reference control, which showed high potency against the SARS-CoV-2 receptor binding domain (RBD) in an in vitro study. The five compounds (CID:1, CID:2, CID:3, CID:4, and CID:5) exhibited remarkable binding affinities (CID:1, − 8.9; CID:2, − 8.7; and CID:3, 4, and 5, − 8.5 kcal/mol) compared to the control compound (− 6.2 kcal/mol). MD simulations over a period of 200 ns further corroborated that certain interactions occurred with the five compounds and the nonalkaloidal compounds retained their positions within the RBD active site. CID:2, CID:4, and CID:5 demonstrated high stability and less variance, while CID:1 and CID:3 were less stable than ceftazidime. The average number of hydrogen bonds formed per timeframe by CID:1, CID:2, CID:3, and CID:5 (0.914, 0.451, 1.566, and 1.755, respectively) were greater than that formed by ceftazidime (0.317). The total binding free energy calculations revealed that the five compounds interacted more strongly within RBD residues (CID:1 = − 68.8, CID:2 = − 71.6, CID:3 = − 74.9, CID:4 = − 75.4, CID:5 = − 60.9 kJ/mol) than ceftazidime (− 34.5 kJ/mol). The drug-like properties of the selected compounds were relatively similar to those of ceftazidime, and the toxicity predictions categorized these compounds into less toxic classes. Structural similarity and functional group analyses suggested that the presence of more H-acceptor atoms, electronegative atoms, acidic oxygen groups, and nitrogen atoms in amide or aromatic groups were common among the compounds with the lowest binding affinities. In conclusion, this in silico work predicts for the first time the potential of using five *R. stricta* nonalkaloid compounds as a treatment strategy to control SARS-CoV-2 viral entry.

## Introduction

Coronavirus (COVID-19) infection is an acute respiratory tract illness induced by severe acute respiratory syndrome coronavirus 2 (SARS-CoV-2). It was initially reported in Wuhan, China, in December 2019^1^. Following its onset, a pandemic of SARS-CoV-2 infection caused disturbances to everyday life and economic activity, and great attempts have been made worldwide to develop effective treatments and vaccines to counteract the pandemic. Notably, the mortality rate of coronavirus disease 2019 (COVID-19) is higher among individuals with obesity and diabetes mellitus and those of older age^2^. Despite the high mortality and morbidity rates associated with COVID-19 infection, no specific therapy is available and the majority of treatment interventions, such as ceftazidime which is used as empirical antibiotic therapy for secondary bacterial infections, are supportive, appear to exert antiviral effect, and based on treating symptoms^3^. According to recent studies, third-generation cephalosporin antibiotics have addressed potent compounds that block the interaction between spike protein and ACE2 by showing their high IC50 values, which support the idea of utilizing ceftazidime as an anti-SARS CoV-2. Nevertheless, most drugs that have been used extensively for the treatment of COVID-19 were repurposing therapeutic candidate drugs, only focusing on a few classic viral sites^4,5^.

The angiotensin-converting enzyme-2 (ACE2) is a transmembrane protein, which is a receptor of the coronavirus' spike protein binding (SARS-CoV2). The distant S1 subunit of the SARS-CoV-2 spike glycoprotein contains the receptor binding domain and is essential to the membrane prefusion state. This glycoprotein is the primary target of antibody neutralization during infection and also the target of treatment and vaccine designs^6^. Earlier viral epidemics, such as SARS and MERS, demonstrated the efficacy of targeting viral entry pathways as a therapeutic strategy^7^. On the one hand, entry inhibitors impede viral transmission between persons; thus, they can be utilized therapeutically and prophylactically. On the other hand, they could be less toxic since they prevent the virus from invading host cells in the first place^3^.

Interest is growing nowadays toward assaying for phytochemicals as natural antivirals. Plants provide us with a range of medicinal compounds that may limit viral reproduction by modulating viral adsorption, attaching to cell surface receptors, preventing virus penetration through the cell membrane, and competing for intracellular signaling pathways. Polyphenols, alkaloids, flavonoids, saponins, quinones, terpenes, proanthocyanidins, lignins, tannins, polysaccharides, steroids, thiosulfonates, and coumarins are all examples of bioactive phytochemicals that have been shown to be effective against viral infections^8^.

*Rhazya stricta* has traditionally been used to treat a variety of illnesses in a wide range of Middle Eastern and South Asian nations*. R. stricta* is known to be a rich source of several potent compounds, including nonalkaloids and alkaloids, which have medicinal applications to treat a variety of conditions, including diabetes, inflammatory diseases, sore throat, helminthiasis, arthritis, infectious diseases, and cancer, and several studies have previously confirmed its folkloric use^9^. Many of these medicinal properties have been validated experimentally by several investigations. Previous studies have proved the antibacterial activities of nonalkaloid extracts derived from *R. stricta* leaves against multi-drug-resistant (MDR) and Extended-spectrum beta-lactamases (ESBLs) bacteria, which make it a drug candidate against several pathogens^10^. The novel natural drug discovery may recognize the new molecular entities that will help even more against variants of SARS-CoV-2.

In the present study, we characterized the *R. stricta* extract using HPLC–MS/MS analysis and conducted a computational study to target the SARS-CoV-2 RBD protein. In addition, molecular docking, dynamic simulations, binding free energy calculations, and ligand bioavailability were performed to determine potential inhibitors. Moreover, we study the structural skeleton similarity of the best-docked compounds to assign their Physicochemical properties.

## Materials and methods

### Collection and preparation of plant samples

*Rhazya stricta* was collected from its natural habitat in the desert; in the Al Gholah region near Asfan Road (21.9684537, 39.2675785), Jeddah Province. A voucher specimen was deposited in the Department of Biological Sciences Herbarium at King Abdulaziz University (number 1150/M/75; collected by N. Baeshen, M. Baeshen, and J. Sabir). The plant material was taken to the laboratory, and the leaves were cut and washed with running water to remove the dust and left to dry in the laboratory at room temperature. A week later, the dry leaves were ground into a fine powder for the extraction of compounds and biochemical analysis. The authors confirm that the experiments performed on the plants in the present study comply with international and national guidelines.

Alkaloids and nonalkaloids were extracted from *R. stricta* as described by^10^. In brief, ten grams of plant material was weighed into a clean volumetric flask, and 20 ml of absolute ethanol (99%) was added. The mixture was allowed to sit in a refrigerator (4 °C) for two days. The ethanol was removed by placing the mixture over Whatman filter paper (0.45 µm) and drying the plant material with nitrogen gas. After that, 5 g of plant material was transferred to a clean volumetric flask, and 40 ml of 1 mol/L HCl and 40 ml of HPLC-grade chloroform were added. The chloroform layer was collected and filtered through a PTFE disk filter and then transferred to an LC vial to analyze the nonalkaloids. For the alkaloids, sodium hydroxide was added to the plant mixture to adjust the pH, and then 40 ml of HPLC-grade chloroform was added. The chloroform layer, which contained the alkaloids, was filtered through a PTFE disk filter and transferred to a liquid chromatography vial for analysis.

### HPLC–MS/MS analysis and data processing

This study was performed using an HPLC–MS/MS system that included an ACQUITY UPLC I-Class (Waters Technologies, USA) instrument coupled to a 6500 Qtrap (AB Sciex, Canada). Chromatographic separation was performed using a Zorbax XDB C18 column (2.1 × 150 mm, 3.5 µm) with a temperature maintained at 40 °C, a flow rate of 300 µL/min and an injection volume of 10 µL. Solvents A (0.1% formic acid in HPLC grade water) and B (0.1% formic acid in HPLC grade acetonitrile) were used as the mobile phases. The linear elution gradient was as follows: 2% B (from 0 to 2), 95% B (from 2 to 24), 95% B (held for 2 min), and 4 min of equilibration. The electrospray ionization mass spectrometry (ESI–MS) data were collected in positive mode (ES+) with an electrode voltage of 5500 V, a declustering potential (DP) of 90 V, collision energy of 30 V, and an input potential of 10 V. Nitrogen was used as the nebulizer gas and curtain gas at 30 psi. Mass spectrometry (MS) spectra were acquired in the mass range of 100–900 m/z, and a scan rate of 1000 was used to search for enhanced production. For MS–MS data collection, the acquisition rate was set to 1 spectrum per second with a scan range of 50–1000 m/z in automode according to^11^.

Upon completion of data collection, the HPLC–MS data files were downloaded in wiff format and then converted to Mzml using MSConvert (ProteoWizard 3.0.20270)^12^. Mzmine (version 2.53) software was used to analyze the results^13^ (https://github.com/mzmine/mzmine2/releases/tag/v2.53). Following data import into Mzmine2, a minimum intensity cutoff of 1,000 was used, and the retention time was set to a tolerance of 0.2 min. Then, the adjusted peaks were compiled into a single mass list to enable detection and comparison. The identification process was performed using the SIRIUS platform coupled with CSI:FingerID for molecular structure identification^14–16^.

### Ligands and protein preparation

Hundreds of nonalkaloid compounds were identified in *R. stricta* by HPLC and MS/MS analyses. The structures of the *R. stricta* nonalkaloid compounds were discovered through HPLC analysis, downloaded from the PubChem database (https://pubchem.ncbi.nlm.nih.gov/) as SDF files, and then converted into PDB files using PyMOL (Schrödinger, LLC)^17,18^. The AutoDock 4.2 graphical interface^19^ was used to prepare the ligands by adding the computed Gasteiger charge and merging the nonpolar hydrogen atoms. Aromatic carbon rings were also detected to set up a torsion tree. The compound files were saved in pdbqt format for further screening. The crystal structure of the SARS-CoV-2 spike receptor-binding domain (RBD) complexed with ACE2 was retrieved from the RCSB (https://www.rcsb.org) with PDB ID 6m0j^21^, consisting of 193 amino acids for the RBD and 596 amino acids for ACE2 with a resolution of 2.45 Å. The RBD was prepared by deleting the ACE2 atom coordinates and removing ligands, water, and ionic metals from the complex structure. Nonpolar hydrogen atoms were merged, and Kollman charges were added to the RBD. The prepared protein was saved as a pdbqt file.

### Virtual screening

AutoDock Vina is an open-source program that predicts the binding positions of small molecules into the cavity of interest of a target protein in addition to the small molecule binding affinities^22^. The grid box was concentrated with dimensions (50X, 64Y, 22Z) and a grid point of 0.375 Å on the interface of the RBD, which contains the involved residues in connection with the ACE2 receptor (K417, G446, Y449, Y453, L455, F456, A475, F486, N487, Y489, Q493, G496, Q498, T500, N501, G502, Y505). AutoDock Vina was run using an in-house Python script, which prompted the system to the configuration file containing the screening parameters. We set the exhaustiveness of the search to 8, and 9 conformation modes were generated for each compound.

### Molecular docking

The best-docked compounds (~ 60), which had the lowest binding affinities and RMSD values of 0 Å into the RBD interface, were subjected to analysis by ACD/ChemSketch to check for tautomeric forms^23^. Geometry optimization was also conducted using Avogadro software, which has an auto-optimization tool^24^. Structure optimization and energy minimization were performed by the universal force field (UFF) and the steepest descent algorithm. To find accurate conformations and positions, we redocked the ligand–protein complexes using AutoDock Vina with the grid box dimensions listed above the exhaustiveness of the search set to 30. Finally, we concentrated the grid box on the lowest binding affinity position of the best 5 compounds and redocked the complexes. To compare our results with those of a drug that has been confirmed to show in vitro anti-SARS-CoV-2 spike protein activity, we found that the antibiotic ceftazidime, which is used therapeutically to treat bacterial pneumonia, revealed the highest potency among several compounds, with an inhibition rate of approximately 80% and an IC50 of 28 µM^25^. Biovia Discovery Studio Visualizer was used to plot the compounds into the pocket residues and PyMOL^17,26^.

### Molecular dynamics simulations

MD simulations were performed using the Groningen Machine for Chemical Simulations (GROMACS) 2020.3^27^. The procedure was conducted as described^28^. To create the structural topology, the compound-protein complex coordinates were separated into two PDB files. The protein force field parameter was generated using the March 2019 version of Chemistry at Harvard Macromolecular Mechanics (CHARMM 36)^29^, whereas the ligand was converted to Mol file format using Avogadro^24^, and the force field parameters were generated using the official CHARMM General Force Field server (CGenFF: 30. The system was contained within a dodecahedron box with periodic boundary conditions. The box was then solvated using a three-site transferable water model (TIP3P)^31^, which was subsequently neutralized with Na and Cl ions. A 5,000-step structural optimization using the steepest descent algorithm followed by a 100 ps equilibration in the NVT and NPT ensembles utilizing a V-rescale thermostat^32^ and a Berendsen barostat^33^ for temperature and pressure coupling were carried out to minimize system energy. The van der Waals and electrostatic interaction cutoffs were fixed at 1.2 nm, and long-range electrostatic interactions were calculated using the particle mesh Ewald method^34^. The temperature and pressure were maintained at 300 K and 1 bar for the production run. A V-rescale thermostat^32^ with a time constant of 0.1 ps was used to regulate the temperature, while a Parrinello-Rahman barostat^35^ with a time constant of 1 ps and compressibility of 4.5 × 10^–5^ bar-1 was employed to the control pressure. The simulation was run for 200 ns, and every 10 ps, the energy and coordinates of the trajectories were recorded. The stabilities of the resultant trajectories were then analyzed using the root mean square deviation (RMSD), the root mean square fluctuation (RMSF), the hydrogen bond number, and the final trajectory. The plotting tool Xmgrace was utilized to plot the simulation data^36^, and PyMOL was used to visualize the obtained trajectories^17^.

### Binding energy calculations

Binding free energy calculations for all docked complexes were conducted using the Molecular Mechanics/Poisson-Boltzmann Surface Area (MM/PBSA) approach in the g_mmpbsa module. The g_mmpbsa scripts utilized were widely sourced from the GROMACS machine and APBS packages to integrate the molecular dynamics simulation trajectories with the binding energy calculations. A total of 500 snapshots of the complex trajectories were obtained. Electrostatic interactions, van der Waals interactions, polar salvation energy, and nonpolar solvation energy were calculated using the g_mmpbsa module^37,38^.

### Drug-likeness analysis

To investigate the oral bioavailability of the top 5 compounds, Lipinski's rule of five^39^ was applied, which considers the following parameters: molecular weight, lipophilicity, number of hydrogen bond donors, and number of nitrogen and oxygen atoms. To predict the drug-likeness profile of the compounds with the lowest binding affinities from the docking results, the PDB files of the compounds were submitted to SwissADME, which is a webserver that calculates the physiochemical properties of compounds and applies drug-likeness parameters^40^. Moreover, the results were compared to the injectable antibiotic ceftazidime.

### Toxicity risk prediction

ProTox-II is a website that predicts the acute oral toxicities of small molecules and places them into classes according to the globally harmonized system of classification and labelling of chemicals (GHS)^41^. Canonical Smiles of the compounds were obtained from the PubChem database and inputted into the webserver. The compound toxicity classes and LD50 predictions were generated as output results.

### Structural skeleton similarity analysis

To find the common structural skeleton similarity of the compounds of interest, we utilized DataWarrior software, which is a multipurpose chemical data visualization and analysis tool that is interactive and chemistry-aware^42^. Forty compounds with a 7.1 (kcal/mol) binding affinity cutoff were submitted to DataWarrior for structure analysis. We counted and analyzed the presence of 8 structural parameters: aromatic carbon atoms, carbo rings, electronegative atoms, H-acceptor atoms, H-donor atoms, heterorings, ring closures, and rotatable bonds.

## Results and discussion

### HPLC–MS/MS analysis

**Figure 1:**
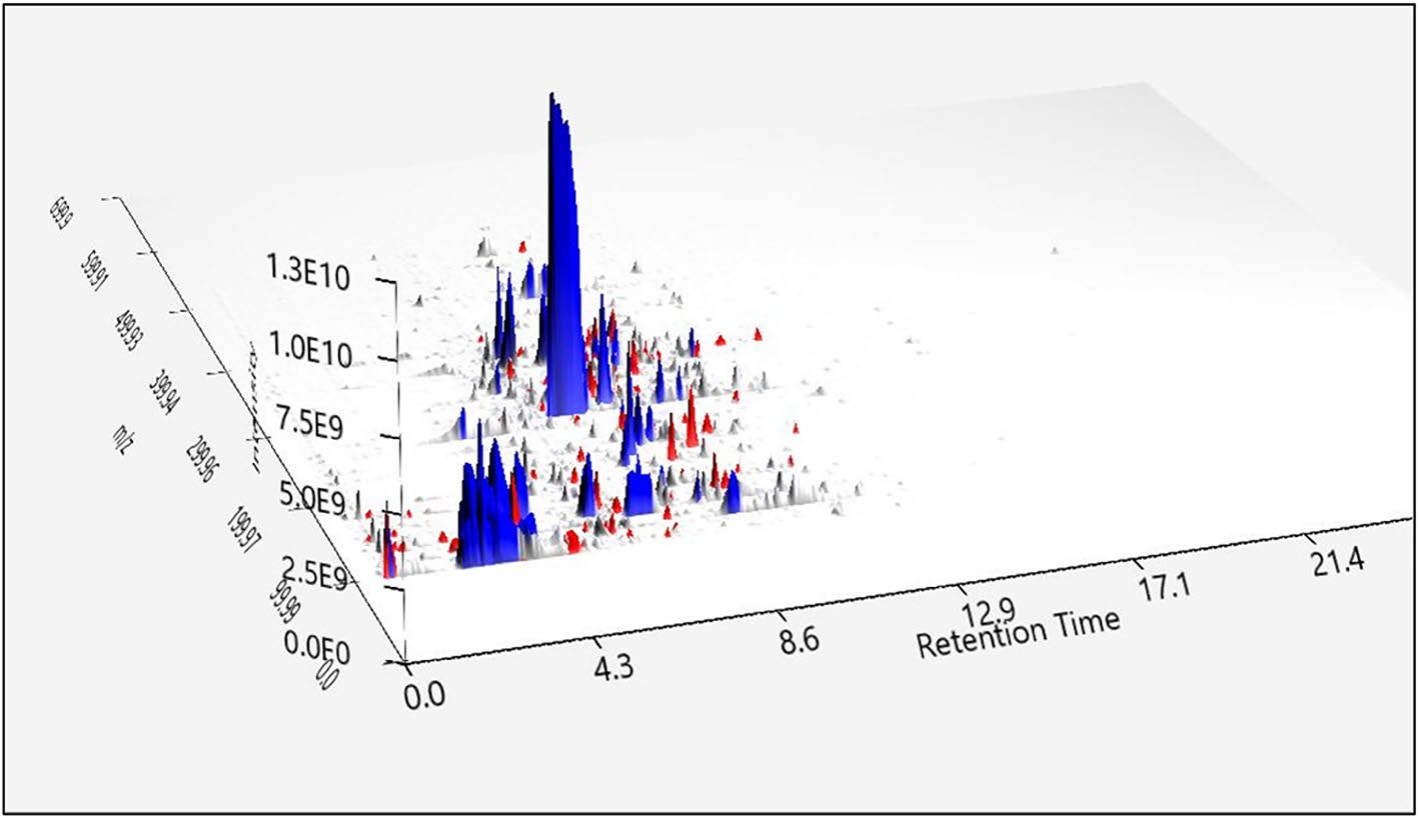
3D chromatogram of the representative extracts using 3D visualizer. RED, alkaloid extract; BLUE, nonalkaloid extract. The X-axis shows the retention time, the Y-axis shows the m/z value, and the Z-axis shows the intensity.

The nonalkaloids from *R. stricta* were isolated using HPLC. Separation using HPLC confirmed the existence of nine nonalkaloid compounds and seven additional alkaloids as minor components (Fig. 1).

### Virtual screening and molecular docking

**Figure 2:**
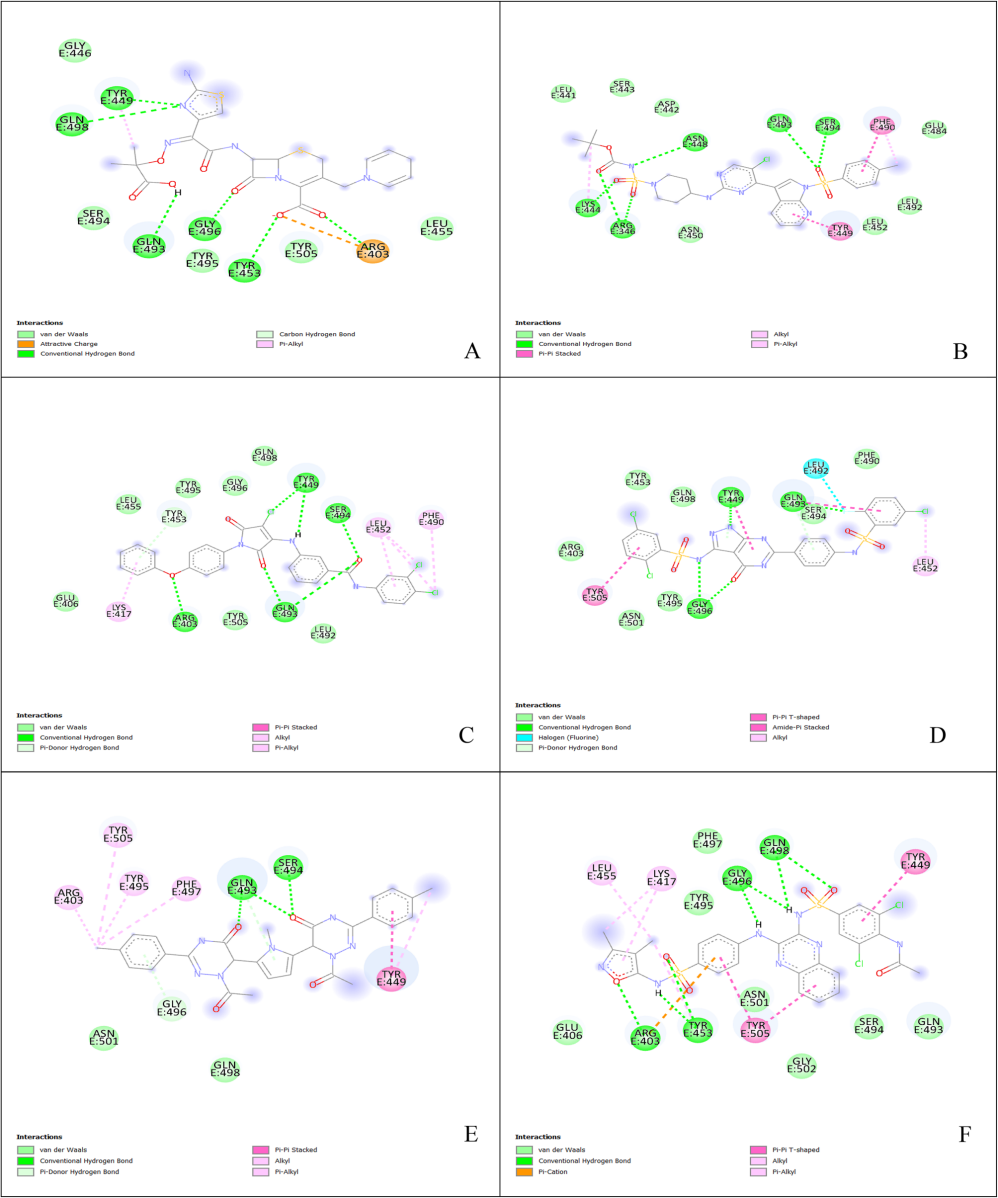
Redocking positions of the selected compounds into SARS-CoV-2 RBD protein pocket generated by AutoDock Vina. (**A**) Ceftazidime. (**B**) CID:1. (**C**) CID:2. (**D**) CID:3. (**E**) CID:4. (**F**) CID:5.

**Figure 3:**
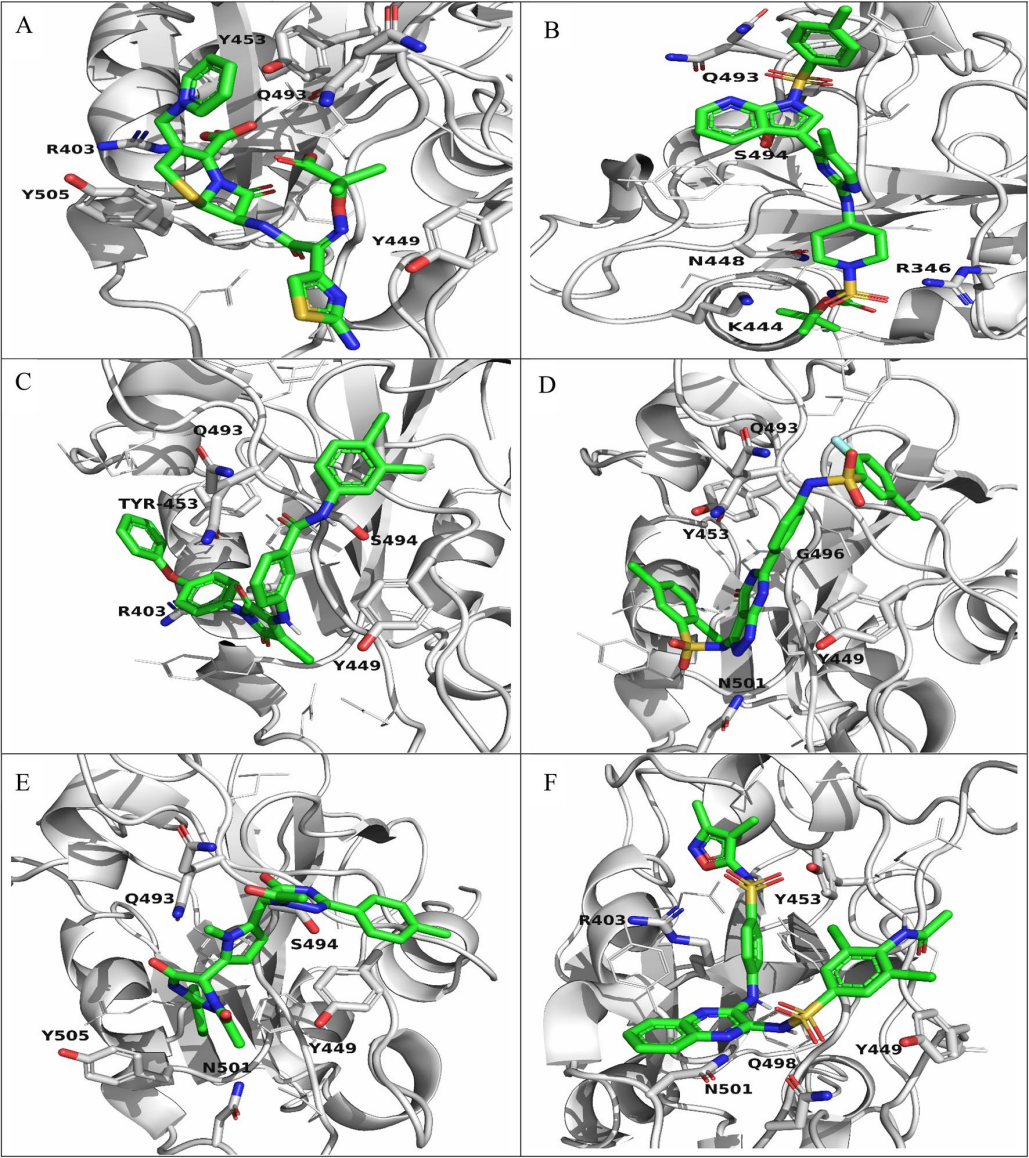
3D visualization showing the redocking poses of the selected compounds and ceftazidime. The white cartoons represent the RBD, and interposes with white sticks represent the contacted residues. The ligands are represented as green sticks. (**A**) Ceftazidime. (**B**) CID:1. (**C**) CID:2. (**D**) CID:3. (**E**) CID:4. (**F**) CID:5.

Virtual screening is a fast-scanning method that can dock a library of ligands into the active site of the protein of interest and reduce the library by eliminating those with the highest binding energies. In our study, after we screened the compounds obtained from the phytochemical analysis, the compounds with the lowest binding affinity were optimized, redocked, and prepared for further analysis. The involved residues and number of bonds formed by the 5 best compounds (Table 1) and the compound control ceftazidime are presented in Table 2. Compound **CID:1** demonstrated a binding affinity of − 8.9 (kcal/mol) and formed six conventional hydrogen bonds with RBD interface residues ARG346, ASN448, LYS444, GLN493, and SER494. In addition, four hydrophobic interactions were observed of the types pi—pi stacked, pi—alkyl, and alkyl in contact with PHE490, TYR449, and LYS444 residues. Compound **CID:2** showed a binding affinity of − 8.7 (kcal/mol) and formed seven hydrogen bonds with residues TYR449, GLN493, SER494, ARG403, and TYR453. Moreover, six hydrophobic interactions of the types pi—pi stacked, pi—alkyl and alkyl involved with the SARS-CoV-2 RBD residues PHE490, LEU452, and LYS417. Compounds **CID:3, CID:4,** and **CID:5** showed binding affinities of − 8.5 (kcal/mol) with various numbers of hydrogen bonds and other interactions. Compound **CID:3** formed six hydrogen bonds with the active site residues TYR449, GLN493, and GLY496. In addition, four hydrophobic interactions of the types T-shaped pi-pi, amide pi-stacked, alkyl interactions with TYR449, TYR505, LEU452, and GLN493, and two halogen bonds with GLN493 and LEU492. Compound **CID:4** formed five hydrogen bonds with the RBD binding site residues GLN493, SER494, and GLY496 and six hydrophobic interactions of the types—pi–pi stacked, pi—alkyl, and alkyl with key residues ARG403, TYR449, PHE497, TYR505, and TYR495. Compound **CID:5** formed seven hydrogen bonds with residues ARG403, TYR453, GLY496, and GLN498 and seven hydrophobic interactions of the types T-shaped pi–pi, pi—alkyl, and alkyl with residues TYR449, TYR505, LYS417, and LEU455. In addition, one pi – cation electrostatic interaction was formed with ARG403. **Ceftazidime** was considered a control, giving a binding affinition of − 6.2 (kcal/mol), and formed six conventional hydrogen bonds with residues ARG403, TYR453, GLY496, TYR449, and GLN498. In addition, attractive electrostatic forces, one pi – alkyl interaction with residues ARG403 and TYR449, and hydrophobic interactions were involved in the ceftazidime-RBD interaction. In addition to the mentioned contacts, the six compounds interacted with the interface residues by van Der Waals forces (Figs. 2 and 3).

**Table 1:** The 2D structures, PubChem CIDs, and formulae of the best docked compounds and the control compound ceftazidime.

CID	PubChem CID	Formula	Structure
1	11585544	C_28_H_32_ClN_7_O_6_S_2_	
2	1677463	C_29_H_18_Cl_3_N_3_O_4_	
3	137148428	C_28_H_14_Cl_3_FN_6_O_5_S_2_	
4	135501155	C_29_H_29_N_7_O_4_	
5	3282882	C_27_H_23_Cl_2_N_7_O_6_S_2_	
6	5481173	C_22_H_22_N_6_O_7_S_2_ (ceftazidime)

**Table 2:** List of the interactions and binding affinities between the selected 5 compounds and SARS-CoV-2 RBD residues found during visualization of the complex structure by discovery studio visualizer.

No.	Binding affinity (kcal/mol)	Residues	Bond distance (Å)	Category	Type
CID:1	− 8.9	ARG346	2.94	Hydrogen Bond	Conventional
		ARG346	3.05	Hydrogen Bond	Conventional
		ASN448	3.37	Hydrogen Bond	Conventional
		LYS444	3.16	Hydrogen Bond	Conventional
		GLN493	3.09	Hydrogen Bond	Conventional
		SER494	3.31	Hydrogen Bond	Conventional
		PHE490	4.10	Hydrophobic	Pi—Pi Stacked
		TYR449	4.76	Hydrophobic	Pi—Pi Stacked
		PHE490	4.34	Hydrophobic	Pi—Alkyl
		LYS444	4.49	Hydrophobic	Alkyl
CID:2	− 8.7	TYR449	2.79	Hydrogen Bond	Conventional
		TYR449	3.19	Hydrogen Bond	Conventional
		GLN493	2.99	Hydrogen Bond	Conventional
		GLN493	3.32	Hydrogen Bond	Conventional
		SER494	3.04	Hydrogen Bond	Conventional
		ARG403	2.80	Hydrogen Bond	Conventional
		TYR453	3.30	Hydrogen Bond	Pi—Donor HB
		TYR453	4.83	Hydrophobic	Pi—Pi Stacked
		LEU452	4.56	Hydrophobic	Alkyl
		LEU452	4.67	Hydrophobic	Alkyl
		LEU452	4.56	Hydrophobic	Pi—Alkyl
		PHE490	4.77	Hydrophobic	Pi—Alkyl
		LYS417	5.15	Hydrophobic	Pi—Alkyl
CID:3	− 8.5	TYR449	3.16	Hydrogen Bond	Conventional
		TYR449	3.71	Hydrogen Bond	Pi—Donor HB
		GLN493	3.27	Hydrogen Bond	Conventional
		GLN493	3.79	Hydrogen Bond	Pi—Donor HB
		GLY496	2.93	Hydrogen Bond	Conventional
		GLY496	3.11	Hydrogen Bond	Conventional
		TYR449	5.06	Hydrophobic	Pi–Pi T-shaped
		TYR449	5.39	Hydrophobic	Pi–Pi T-shaped
		TYR505	5.02	Hydrophobic	Pi–Pi T-shaped
		LEU452	4.99	Hydrophobic	Alkyl
		LEU492	3.62	Halogen	Fluorine
		GLN493	3.27	Halogen	Fluorine
CID:4	− 8.5	GLN493	2.98	Hydrogen Bond	Conventional
		GLN493	3.06	Hydrogen Bond	Conventional
		SER494	3.09	Hydrogen Bond	Conventional
		SER494	3.55	Hydrogen Bond	Pi—Donor HB
		GLY496	3.42	Hydrogen Bond	Pi—Donor HB
		TYR449	4.45	Hydrophobic	Pi—Pi Stacked
		ARG403	4.30	Hydrophobic	Alkyl
		TYR449	5.32	Hydrophobic	Pi—Alkyl
		PHE497	4.71	Hydrophobic	Pi—Alkyl
		TYR505	5.11	Hydrophobic	Pi—Alkyl
		TYR495	5.26	Hydrophobic	Pi—Alkyl
CID:5	− 8.5	TYR453	2.83	Hydrogen Bond	Conventional
		TYR453	2.97	Hydrogen Bond	Conventional
		ARG403	2.89	Hydrogen Bond	Conventional
		GLY496	3.07	Hydrogen Bond	Conventional
		GLY496	3.37	Hydrogen Bond	Conventional
		GLN498	2.73	Hydrogen Bond	Conventional
		GLN498	3.16	Hydrogen Bond	Conventional
		ARG403	4.57	Electrostatic	Pi—Cation
		TYR449	5.55	Hydrophobic	Pi–Pi T-shaped
		TYR505	5.09	Hydrophobic	Pi–Pi T-shaped
		TYR505	5.20	Hydrophobic	Pi–Pi T-shaped
		LYS417	3.99	Hydrophobic	Alkyl
		LEU455	4.86	Hydrophobic	Alkyl
		LYS417	5.22	Hydrophobic	Pi—Alkyl
		LEU455	5.49	Hydrophobic	Pi—Alkyl
Ceftazidime	− 6.2	ARG403	2.95	Hydrogen Bond	Conventional
		ARG403	3.45	Hydrogen Bond	C–H Bond
		TYR453	3.02	Hydrogen Bond	Conventional
		GLY496	3.07	Hydrogen Bond	Conventional
		TYR449	3.07	Hydrogen Bond	Conventional
		GLN498	3.26	Hydrogen Bond	Conventional
		ARG403	5.15	Electrostatic	Attractive
		TYR449	5.00	Hydrophobic	Pi—Alkyl

In addition to the reference compounds, the five compounds formed many bonds with key residues LYS417, TYR453, TYR505, ASN501 GLN493, GLY496, TYR449, GLN498, and LEU455 of the SARS-CoV-2 RBD, which may interrupt the viral host cell recognition process. The 5 phytochemical compounds investigated through molecular docking simulations here revealed significantly better binding energy. In contrast to the reference compounds, these five compounds established networks of hydrophobic interactions that contribute to the binding affinity of the predicted complexes.

### MD simulations and binding free energy calculations

We further used GROMACS 2020.3 to run MD simulations on the five RBD-ligand complexes as well as the control compound on a 200 ns time scale to investigate the dynamic binding interactions and calculate the binding free energies.

#### RMSD and RMSF

**Figure 4:**
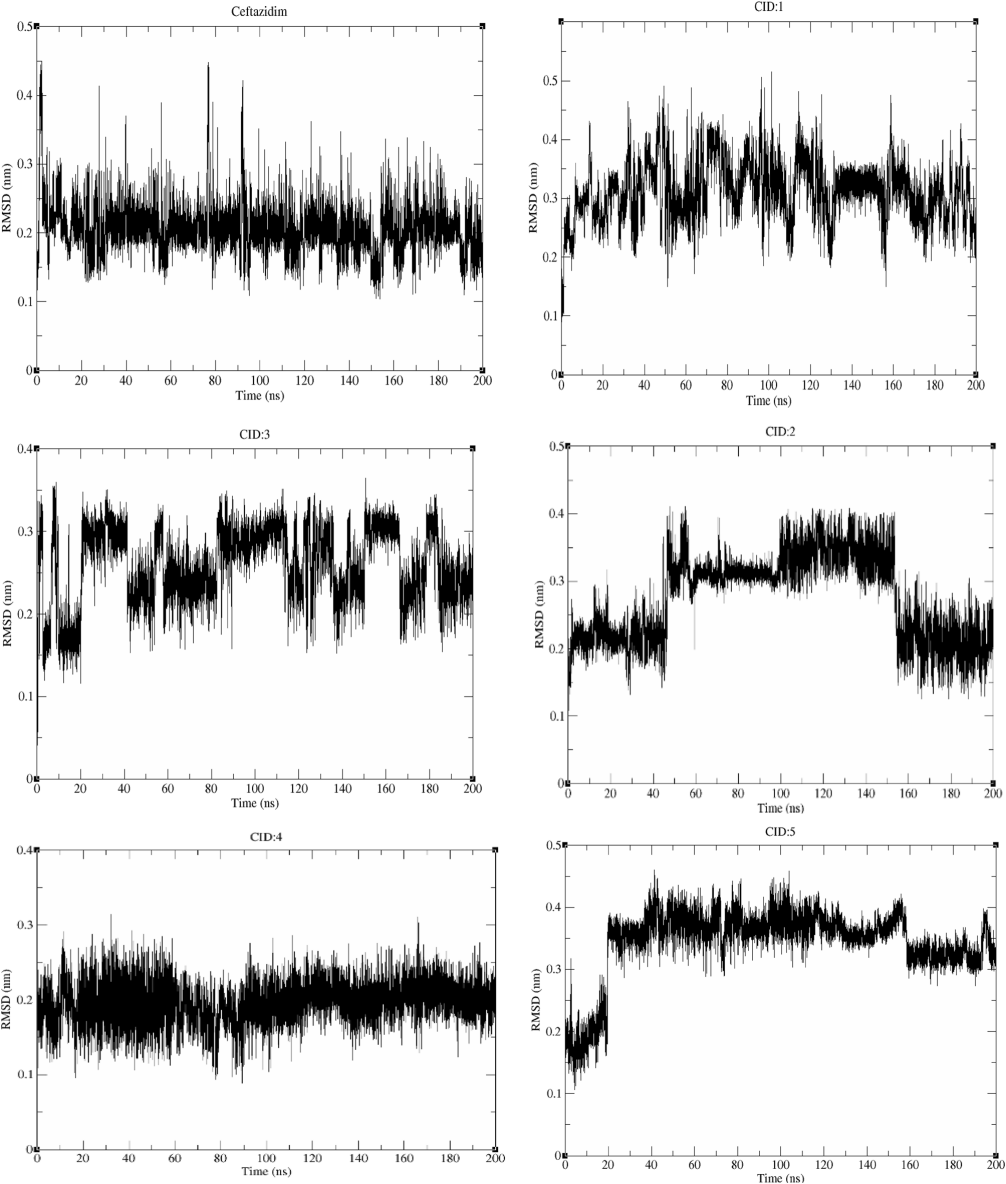
Root mean square deviations (RMSDs) of the ligand–protein complexes generated during the MD simulations.

**Figure 5:**
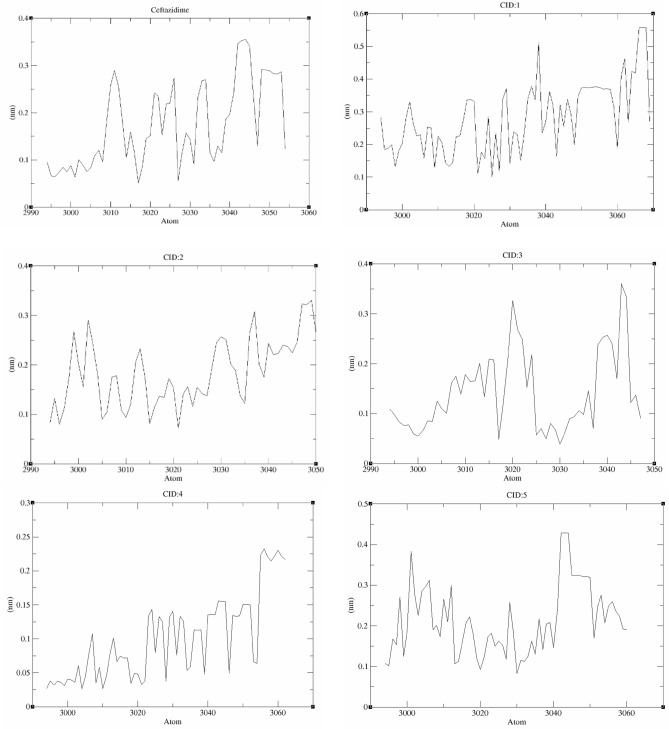
Root mean square fluctuations (RMSFs) of the ligand–protein complexes generated during MD simulations.

The root mean square deviation (RMSD) is a critical measure for analyzing the equilibration of MD trajectories and determining the stability of protein–ligand complex systems during the simulation process. This value was calculated for each compound and compared to that of ceftazidime with respect to the initial pose as a reference frame. All compounds retained their docking position with some deviation (Fig. 4). **Ceftazidime** showed insignificant deviation (~ 0.18–0.27 nm) and high stability with rare major variance. During the MD simulations, compounds **CID:1** and **CID:3** showed acceptable deviations (~ 0.28–0.4 and 0.2–0.35 nm, respectively) and stability with major variance in some frames. Compound **CID:2** exhibited moderate deviations during the first and last 50 ns (~ 0.2–0.26 nm), and higher deviations between 50–150 ns (~ 0.3–0.4 nm) were observed. In addition, compound **CID:2** showed high stability in both poses, and no major variance was observed. Compound **CID:4** showed insignificant deviation (~ 0.15–0.24 nm), high stability and no variance. Although compound **CID:5** had a high deviation (0.34–0.42 nm), it also had high stability with no undesired variance. To understand the behavior of the ligand atoms, the root mean square fluctuation (RMSF) during the MD simulations of the 5 compounds and **ceftazidime** were analyzed. All compounds experienced a certain amount of fluctuation (Fig. 5), and the atoms of **CID:4** fluctuated the least (~ 0.05–0.25 nm) compared with the atoms of the other compounds, indicating that **CID:4** did not undergo major conformational changes. The atoms of **ceftazidime**, **CID:2**, and **CID:3** fluctuated in the range of ~ 0.1–0.35 nm. The RMSF of **CID:1** and **CID:5** reflected the RMSD results, as these two compounds were exposed to conformational changes (~ 0.15–0.55 and 0.1–0.45 nm, respectively).

#### Hydrogen bonds

**Figure 6:**
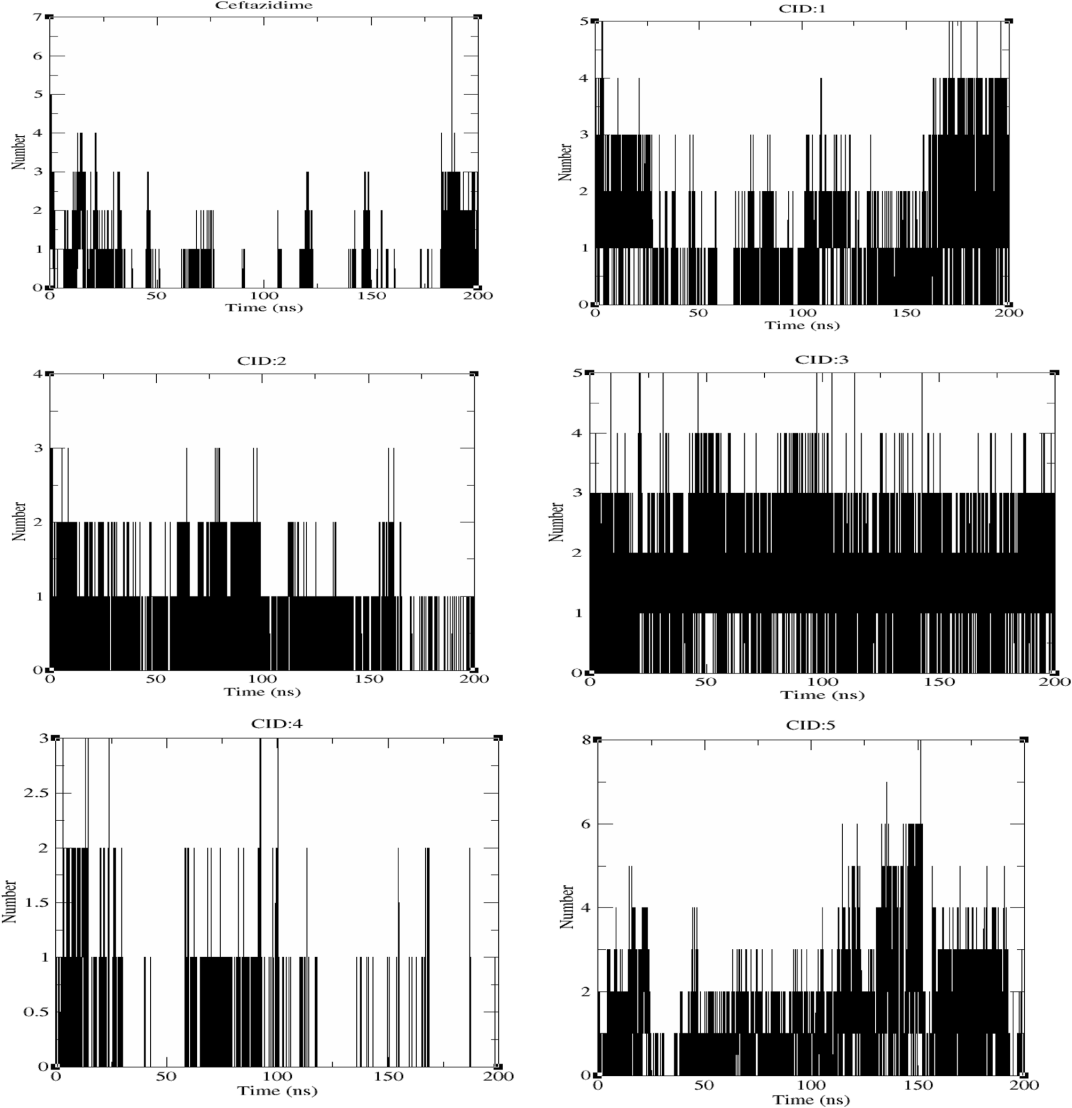
Number of hydrogen bonds in the ligand–protein complexes generated during the MD simulations.

The number of hydrogen bonds that a compound forms with protein residues plays a critical role in enhancing complex stability. The cutoff angle and distance for H-bond analysis were set to 30° and 3.5 Å (Fig. 6). **Ceftazidime** formed 1–7 hydrogen bonds, and the average of 0.317 hydrogen bonds per timeframe was calculated. **CID:1** and **CID:3** showed 1–5 hydrogen bonds bound to the RBD with average numbers of hydrogen bonds per timeframe of 0.914 and 1.566, respectively. **CID:2** and **CID:4** formed 1–3 hydrogen bonds for averages of 0.451 and 0.149 hydrogen bonds per timeframe, respectively. **CID:5** had the highest number of hydrogens (1–8) that formed and an average of 1.755 hydrogen bonds per timeframe.

#### Trajectory

**Figure 7:**
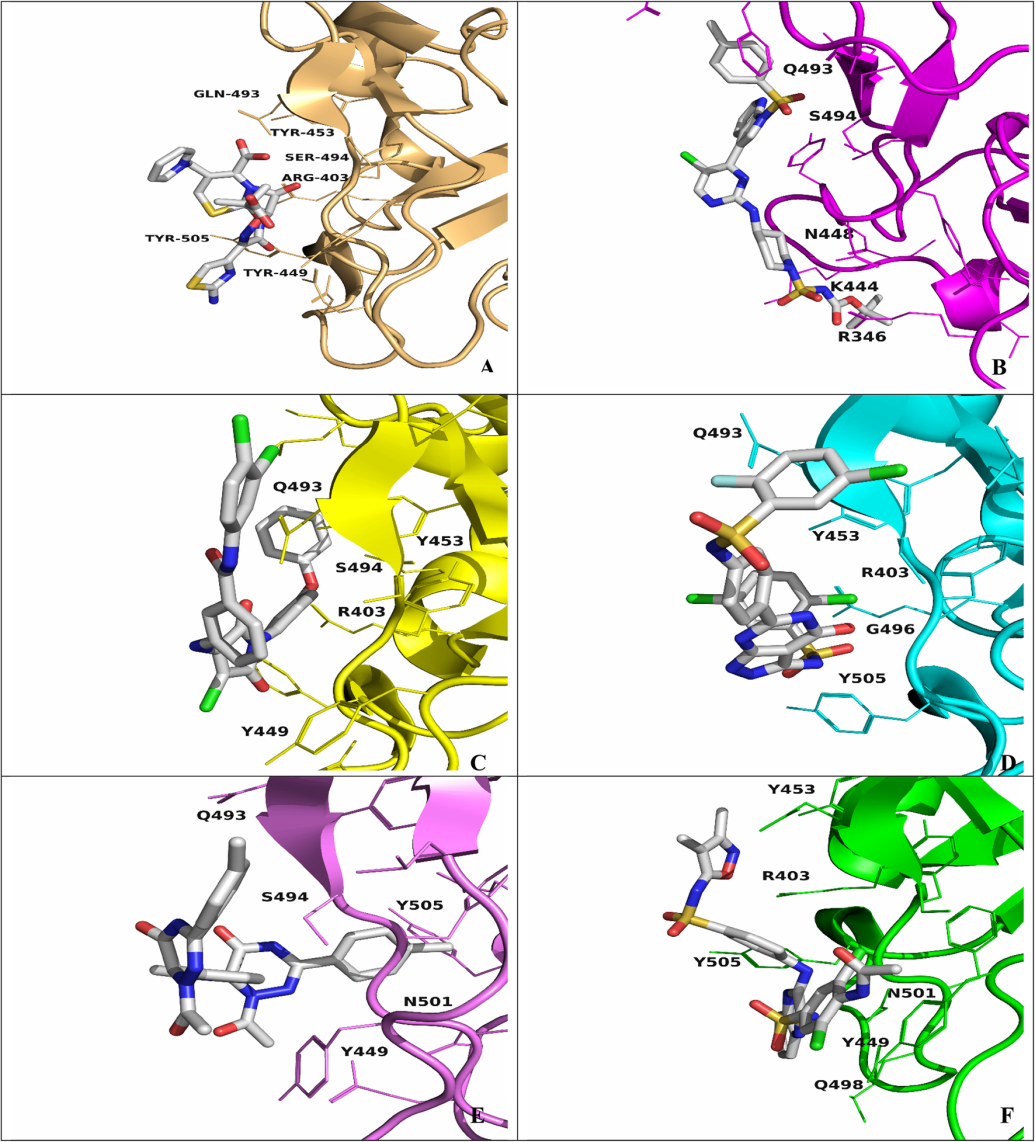
Docked positions of the ligand–protein complexes after MD simulations generated by extracting the last frame of the simulation. (**A**) Ceftazidime. (**B**) CID:1. (**C**) CID:2. (**D**) CID:3. (**E**) CID:4. (**F**) CID:5.

The compound positions and the interacting residues during the MD simulations were visualized by extracting the final frame. In addition to **ceftazidime**, the docked MD simulations poses of compounds **CID:1 CID:2**, **CID:3**, **CID:4**, and **CID:5** were nearly identical to the redocked poses with the exception of **CID:3**, which abandoned its polar interactions with ASN501 to TYR505. Notably, the resulting docked position of **ceftazidime** was compatible with a previous investigation, which revealed that the residues SER494 and TYR505 play critical roles in the binding of SARS-CoV-2 to the hACE2 receptor^23^ (Fig. 7).

#### Binding energy calculations

The MM-PBSA analyses were performed by extracting 500 snapshots of the stabilized frames for the 5 complexes as well as the control complex to calculate the binding energy average. A highly negative binding energy indicates stable binding of a small molecule to a protein. Compared to the RBD-ceftazidime complex (− 34.495 kJ/mol), the resulting binding free energies of the five ligand complexes gave values that were more negative, indicating that these 5 compounds established strong interactions. **CID:4** showed the lowest negative value (− 75.448), followed by compounds **CID:3** (− 74.926 kJ/mol), **CID:2** (− 71.579 kJ/mol), **CID:1** (− 68.788 kJ/mol), and **CID:5** (− 60.865 kJ/mol) (Table 3).

**Table 3:** Average binding energies of the ligand–protein interactions calculated using the g_mmpbsa tool by extracting 500 snapshots from the MD simulation trajectories.

Compound	Binding energy				
	Binding energy (kJ/mol)	SASA energy (kJ/mol)	Polar solvation energy (kJ/mol)	Electrostatic energy (kJ/mol)	van der Waals energy (kJ/mol)
CID:1	− 68.788 ± 35.876	− 15.580 ± 0.973	83.490 ± 36.496	− 9.369 ± 7.338	− 127.330 ± 8.552
CID:2	− 71.579 ± 27.997	− 15.799 ± 1.109	179.500 ± 26.762	− 62.060 ± 10.516	− 173.220 ± 12.079
CID:3	− 74.926 ± 15.849	− 15.799 ± 1.109	174.336 ± 12.529	− 60.002 ± 9.974	− 173.460 ± 11.956
CID:4	− 75.448 ± 12.148	− 15.074 ± 1.034	84.417 ± 14.235	− 20.594 ± 5.837	− 124.197 ± 9.626
CID:5	− 60.865 ± 38.282	− 18.880 ± 1.518	186.375 ± 37.195	− 54.743 ± 12.287	− 173.616 ± 16.981
Control	− 34.495 ± 26.438	− 15.799 ± 1.109	47.016 ± 31.186	− 14.287 ± 12.453	− 51.425 ± 16.130

The MMPBSA-based binding energy values of the identified ligands toward the SARS-CoV-2 RBD reflect that each ligand binds efficiently. This conclusion is further supported by data from other parameters, such as the RMSD, RMSF, number of HBs, and average number of HBs per frame calculated from the MD trajectories.

### Drug-likeness and toxicity predictions

Drug-likeness predictions were conducted using SwissADME to calculate the physiochemical properties and then applying Lipinski’s rule of five for comparison with **ceftazidime**, which is an injectable broad-spectrum antibiotic used to treat bacterial infections including lower respiratory tract pneumonia^43^. Violation of the implemented parameters of Lipinski’s rule of five suggests increased absorption and permeation difficulties if the compounds are administered orally^39^. All compounds as well as the control compounds violated the molecular weight parameter (662.18, 578.83, 643.88, 539.59, 676.55, and 546.6 g/mol for CID:1, CID:2, CID:3, CID:4, CID:5 and ceftazidime, respectively). In addition, the parameter for the number of H-bond acceptors was violated by the investigated compounds with the exception of compound **CID:2**, which had 7 H-bond acceptor atoms (Table 4). In addition to the drug likeness predictions, drug toxicity was predicted using the ProTox-II webserver. The quantities of each substance that resulted in death to 50% of the population (LD50) were determined and categorized according to the globally harmonized system of classification and labelling of chemicals. The LD50 values of compounds **CID:1**, **CID:2**, and **CID:4** (464, 705, and 1300 mg/kg, respectively) were predicted to belong to class 4 (300 < LD50 ≤ 2000). Compound **CID:3** showed less toxicity with an LD50 of 3016 mg/kg and was categorized into class 5 (2000 < LD50 ≤ 5000). **CID:5** was labeled as nontoxic compound with an LD50 of 10,000 mg/kg, and classified as class 6 (LD50 > 5000) (Table 5).

**Table 4:** Molecular weights (MWs), number of oxygen and nitrogen atoms, number of hydrogen bond acceptors (–H), and lipophilicity (LogP) of the selected compounds and the control.

Compound	500 ≤ MW (g/mol)	10 ≤ N or O	5 ≤ NH or OH	4.15 ≤ MLogP
CID:1	662.18	13	2	2.87
CID:2	578.83	7	2	4.11
CID:3	643.88	11	4	3.66
CID:4	539.59	11	2	2.45
CID:5	676.55	11	4	2.65
Ceftazidime	546.6	11	3	0.59

**Table 5:** LD50 values of the selected compounds and their respective classes.

Compound	LD50 (mg/kg)	Class
CID:1	464	4
CID:2	705	4
CID:3	3016	5
CID:4	1300	4
CID:5	10,000	6
Ceftazidime	10,000	6

These results suggest that the bioactive compounds **CID:1**, **CID:2**, **CID:3**, **CID:4**, and **CID:5** have lower gastrointestinal absorption properties compared with ceftazidime. In contrast to **CID:5** and ceftazidime, which were predicted to be nontoxic substances, **CID:1** and CID:2 may possess toxicity risks, and **CID:4** and **CID:3** are associated with less risk.

### Structural skeleton similarity analysis

**Figure 8:**
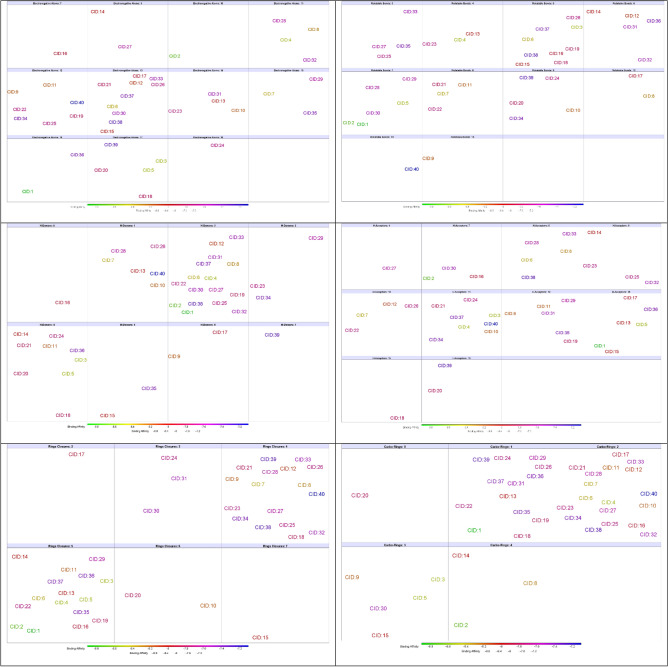

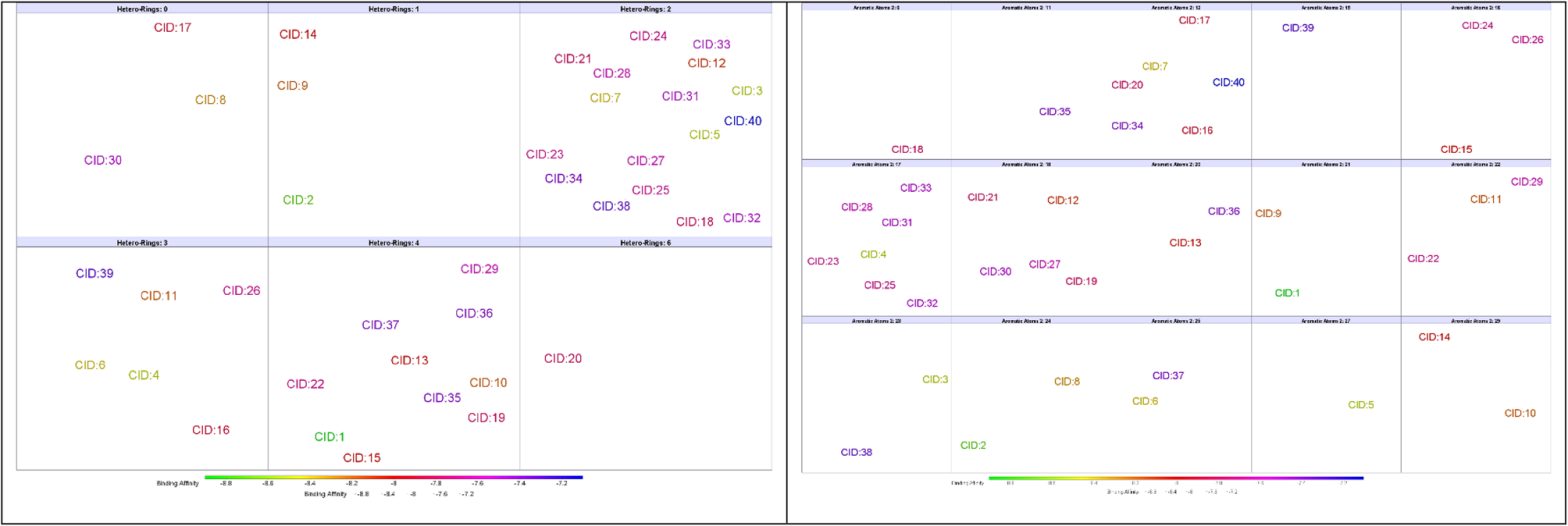
Distribution of the compounds into numeric groups based on their structural skeleton parameters.

The 40 compounds that gave best binding affinities were submitted to DataWarrior software analysis, which was utilized to count and categorize 8 structural skeleton parameters and investigate their similarities (Fig. 8). To define the flexibility of the compounds, we counted the number of intramolecular rotatable bonds and found that 29 of the compounds were composed of 5–9 rotatable bonds, including 7 out of the best 10 compounds. Electronegative atoms (N, O, S, F, Cl) within small molecules play critical roles in forming hydrogen bonds with protein residues; at least 7 of these atoms were counted in **CID:16**, and 28 compounds had 11–15 electronegative atoms. To determine the form of the electronegative atoms, we counted the number of H-bond donors and H-bond acceptors. Twenty-eight compounds contained 2–4 H-donor atoms, including 7 of the best docked compounds. In contrast, at least 4 H-acceptor atoms were counted in **CID:27**, and 28 compounds, including 7 of the best docked compounds, contained 9–13 H-acceptor atoms. Ring closures, which are mostly composed of carbon atoms involved in hydrophobic and electrostatic interactions, were counted, and we found 33 compounds containing 4 or 5 rings, including the best 9 compounds. To determine the form of the ring closures, we counted both carbo- and heteroring closures. Among the carbo rings, we found 29 compounds containing 1–2 rings. In contrast to the carborings, 33 compounds contained 2–4 heterorings, including 7 of the best 10 compounds. Finally, we counted the number of aromatic carbon atoms, and the 40 compounds were distributed among 15 categories. Twenty-seven compounds were in the range of 17–27 aromatic atoms, including 8 of the ten best compounds.

After we characterized and analyzed the 40 compounds that gave an affinity binding score of − 7.1 (kcal/mol) or less, we found no direct or inverse relationship between the parameters that we investigated and the binding affinities of the compounds. In addition, the desired range and cutoff of some of the parameter combinations could lead to an increase in the binding affinity of small molecules to the SARS-CoV-2 RBD, such as at least 7 electronegative atoms, 5–9 rotatable bonds, 4–2 H-donor atoms, at least 7 H-acceptor atoms, 1–3 carbo rings, and 2–4 hetero rings.

## Conclusion

Our study aimed to find a prospective drug against SARS-CoV-2 and examine the similarities among the investigated compounds by utilizing compounds derived from the medicinal plant *R. stricta*. Applying a virtual screened approach, we identified five compounds from *the R. stricta nonalkaloid extract.* In comparison to the reference control (ceftazidime), the lead compounds exhibited remarkable binding affinities and strong interactions with key residues of the SARS-CoV-2 spike protein. The findings of this in silico study suggest that these compounds can be considered potential antiviral drugs to treat SARS-CoV-2 by interfering with the viral ACE2 recognition process. However, more experimental validation is required to confirm the antiviral activity of the selected compounds against the SARS-CoV-2 RBD. The two main outcomes from the structural skeleton analysis could help researchers design or narrow their search to find bioactive compounds targeting the SARS-CoV-2 RBD: electronegative atoms are preferable in the H-acceptor form, and ring closures are preferable in heteroatom form. However, a large compound population and more structural skeleton parameter analyses are required to reveal more recommended characteristics and confirm our findings.

## Supplementary Information

Supplementary Information.
