# Supplementary material for: In silico screening of some compounds derived from the desert medicinal plant Rhazya stricta for the potential treatment of COVID-19
Source: Sci Rep. 2022 Jul 1;12:11120. doi: 10.1038/s41598-022-15288-2 (PMC9247940; doi:10.1038/s41598-022-15288-2)
Supplement: Supplementary file 1 — Supplementary Information. [file 41598_2022_15288_MOESM1_ESM.pptx]

## Slide 1
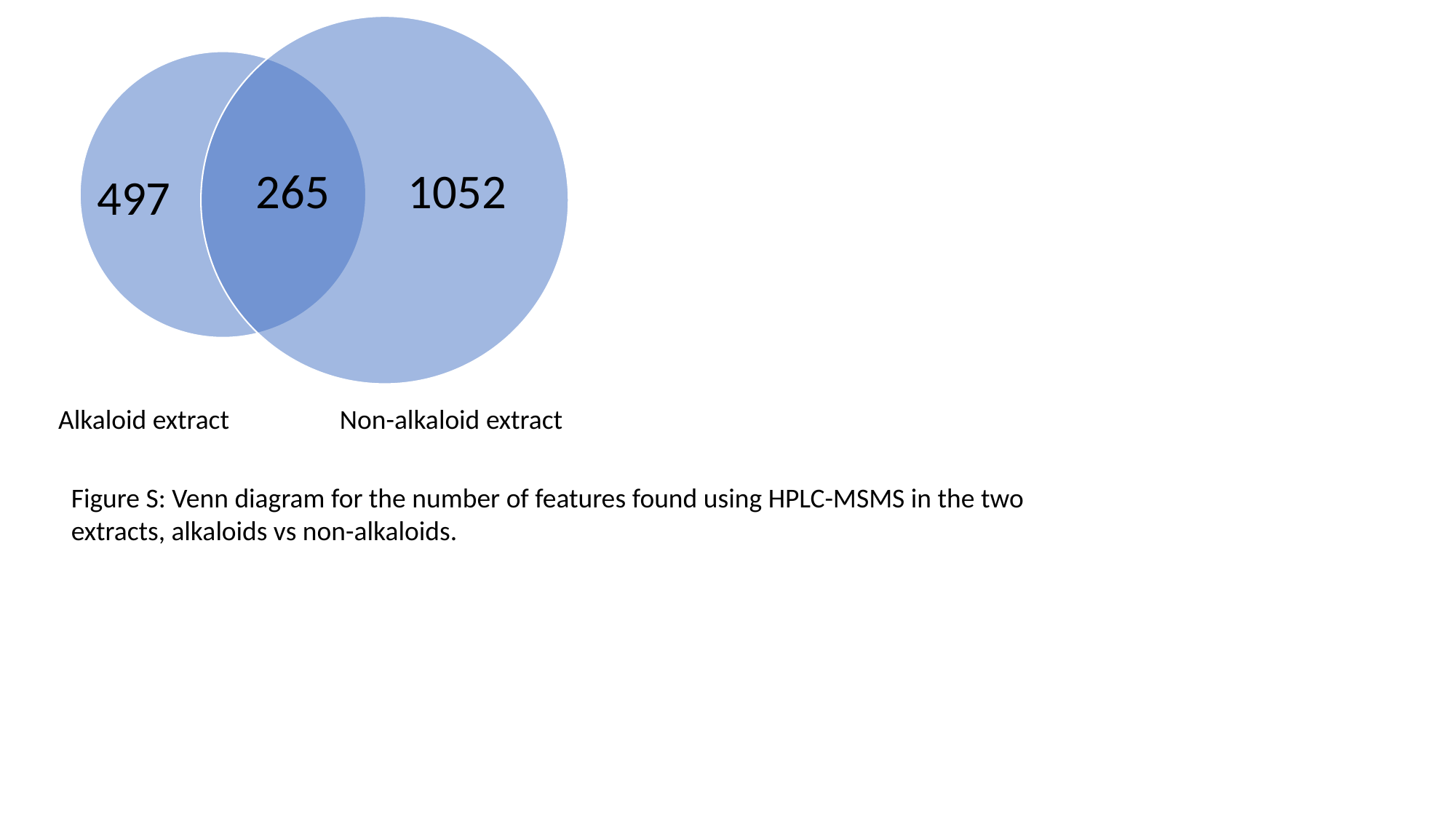

265
1052
497
Alkaloid extract
Non-alkaloid extract
Figure S: Venn diagram for the number of features found using HPLC-MSMS in the two extracts, alkaloids vs non-alkaloids.

## Slide 2
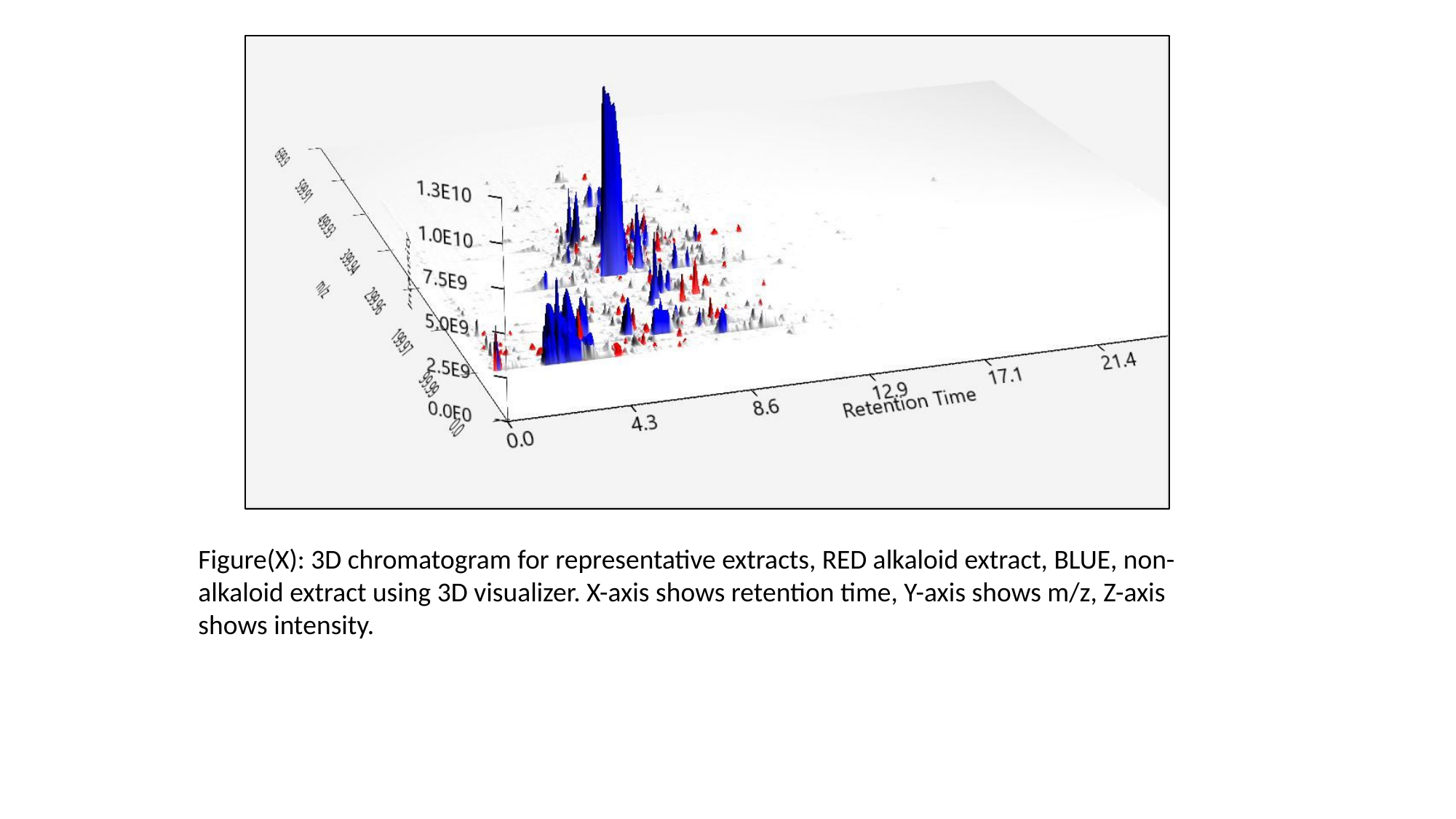

Figure(X): 3D chromatogram for representative extracts, RED alkaloid extract, BLUE, non-alkaloid extract using 3D visualizer. X-axis shows retention time, Y-axis shows m/z, Z-axis shows intensity.
